# High throughput screening of cytokines, chemokines and matrix metalloproteinases in wound fluid induced by mammary surgery

**DOI:** 10.18632/oncotarget.4828

**Published:** 2015-08-10

**Authors:** Dan Wang, Kebang Hu, Ningning Gao, Hao Zhang, Yanlin Jiang, Caigang Liu, Shouyu Wang, Zuowei Zhao

**Affiliations:** ^1^ Breast Disease and Reconstruction Center, Breast Cancer Key Lab of Dalian, The Second Hospital of Dalian Medical University, Dalian, China 116023; ^2^ Department of Urology, First Hospital of Jilin University, Changchun, China 130021; ^3^ Ultrasonic Diagnosis Department, The First Hospital of China Medical University, Shenyang, China 110001; ^4^ Department of Surgery, the first Hospital of Dalian Medical University, Dalian, China 114000

**Keywords:** breast cancer, wound fluid, proliferation

## Abstract

**Objective:**

To clarify the composition of wound fluid (WF) and investigate the impact of WF on breast cancer cell lines.

**Methods:**

The proliferation and migration of WF-treated breast cancer cells MDA-MB-231 and MCF-7 were assessed with colony formation test, MTT cell proliferation test and scratch wound test. The quantitative profiles of WF were analyzed using Bio-Plex Pro kits.

**Results:**

The proliferation and migration of WF-treated breast cancer cells were significantly higher than that of untreated cells. Fifteen cytokines, 29 chemokines and 9 matrix metalloproteinases (MMPs) were assessed in WF. The concentrations of these factors were influenced by post-surgery days, neoadjuvant chemotherapy (NAC), TNM stage, pathological type and molecular subtype. The WF harvested from patients underwent NAC showed significant higher profiles of interleukin-1β (IL-1β), IL-4, IL-6, IL-17F, IL-21, IL-23, IL-25, IL-31, Interferon γ (IFNγ), CD40 ligand (CD40L), tumor necrosis factor α (TNFα), CXCL1, CXCL2, CXCL5, CCL3, CCL7 and CCL20.

**Conclusions:**

Surgery-induced WF promotes the proliferation and migration of breast cancer cells. The composition of WF is influenced by various clinical features and provides potential therapeutic targets to control local recurrence and tumor progression.

## INTRODUCTION

Breast cancer is the most common malignancy in women and late-stage diseases show a high mortality rate. In 2008, 1, 380, 000 new occurrences of breast malignancies were diagnosed worldwide, with 458, 400 cases of cancer-related deaths [1]. Although various breast cancer treatments, including surgery, radiotherapy, endocrine therapy, targeted therapy, and cytotoxic therapy, have significantly improved patient survival in the past decades, cancer metastasis and relapse are still commonly seen. More than 40% of the breast cancer patients develop tumor recurrence after they have received comprehensive anti-cancer treatments [2].

To date, surgery serves as one of the standard treatments for breast cancer; however, the adverse impact of surgery remains controversial, given that surgical intervention may change certain tumor microenvironment, which further modifies the growth kinetics of breast cancer cells. Previous studies showed that tumor growth increased at the corresponding site of the surgical wounds [3, 4]. As reported, 90% of local recurrences occur at the same quadrant of the primary cancer [5]. Compared to untreated patients with a recurrence peak at 4 to 5 years after initial operation, surgery induced a 2 to 3 years recurrence peak after patients underwent mastectomy [6]. Surgery is also likely to stimulated the metastasis via the crosstalk among the host cells, the primary tumor and circulating tumor cells or metastatic tumor cells, which may present at the time of surgery, as already demonstrated in animal models and reported in clinical researches [7, 8]. Moreover, a study characterizing micrometastases demonstrated that in some cases, primary tumors produced angiogenesis inhibitor factors, and therefore, primary tumor removal caused a switch from micrometastatic foci to angiogenic phenotypes, resulting in increased metastases [9].

Several studies revealed that wound fluid (WF) derived at surgical site acted as a stimulative factor in tumor progression. Licitra *et al*. found that epidermal growth factor-like (EGF-like) growth factors in WF derived from surgically resected head and neck squamous cell carcinomas (HNSCCs) induced proliferation of squamous carcinoma cell lines by promoting epidermal growth factor receptor (EGFR) expression and activating EGFR pathway [10]. Segatto and colleagues reported that surgery-induced WF promoted stem-like and tumor-initiating features of breast cancer cells via STAT3 signaling [11]. Moreover, quantitative molecular diagnosis including carcinoembryonic antigen (CEA) and cytokeratin-19 (CK-19) assays targeting cancer cells in axillary WF revealed that CEA and CK-19 were predictor for locoregional recurrence in breast cancer patients with mastectomy [12].

Although WF is rich in biological factors, the expression of these factors and how they interact with tumor cells have not been clearly characterized. As we all know, wound healing itself elicits a range of inflammatory responses, while these responses may play pivotal roles in cancer development, including tumorigenesis, tumor cells growth, proliferation, angiogenesis, invasion and metastasis [13, 14]. Cytokines, especially interleukin-6 (IL-6) family, are now recognized as important mediators linking inflammation and cancer, and are potential therapeutic and preventive targets as well as prognostic factors [15]. IL-6 family is highly up-regulated in many cancers including breast cancer and is considered as one of the most important cytokine families contributing to cancer development [15]. Chemokine, known as a kind of small molecular basic protein that recruits and activates leukocytes, plays an essential role in inflammatory responses. Tumor cells can change the chemotactic reaction of leukocyte to chemokine and evade the immune attack by inactivating chemokine. It has been proved that CXCL1, CXCL2, CXCL5, CXCL6, CXCL12, CCL2 and CCL11 (Glu-Leu-Arg motif (ELR) negative chemokines) are angiogenic, whereas CXCL9, CXCL10 and CXCL11 (ELR positive chemokines) are angiostatic [16, 17]. Saji *et al*. also reported that CCL2 (monocyte chemoattractant protein, MCP-1) could be detected in 51% of primary breast cancer, and was closely related to tumor-associated macrophages (TAMs), microvessel density (MVD) and matrix metalloproteinase (MMP) [18]. MMPs, serving as one of the most important extracellular matrix (ECM) metabolic enzymes, can degrade macromolecular components of ECM, which subsequently make a contribution to tumor infiltration, metastasis and angiogenesis. Furthermore, some chemokoines significantly change tumor behavior by regulating MMPs.

Given the evidence provided in previous publications, we hypothesize that WF can modify tumor progression by interacting with chemokines, cytokines and MMPs. In the current study, we aimed to investigate the effect of WF on the proliferation and migration of breast cancer cells and characterize the levels of the cytokines, chemokines and MMPs in breast surgery induced WF.

## RESULTS

### Effects of WF on breast cancer cells proliferation

The proliferation of MCF-7 and MDA-MB-231 was assessed *in vitro* by performing colony formation assay and MTT proliferation assay.

**Figure 1:**
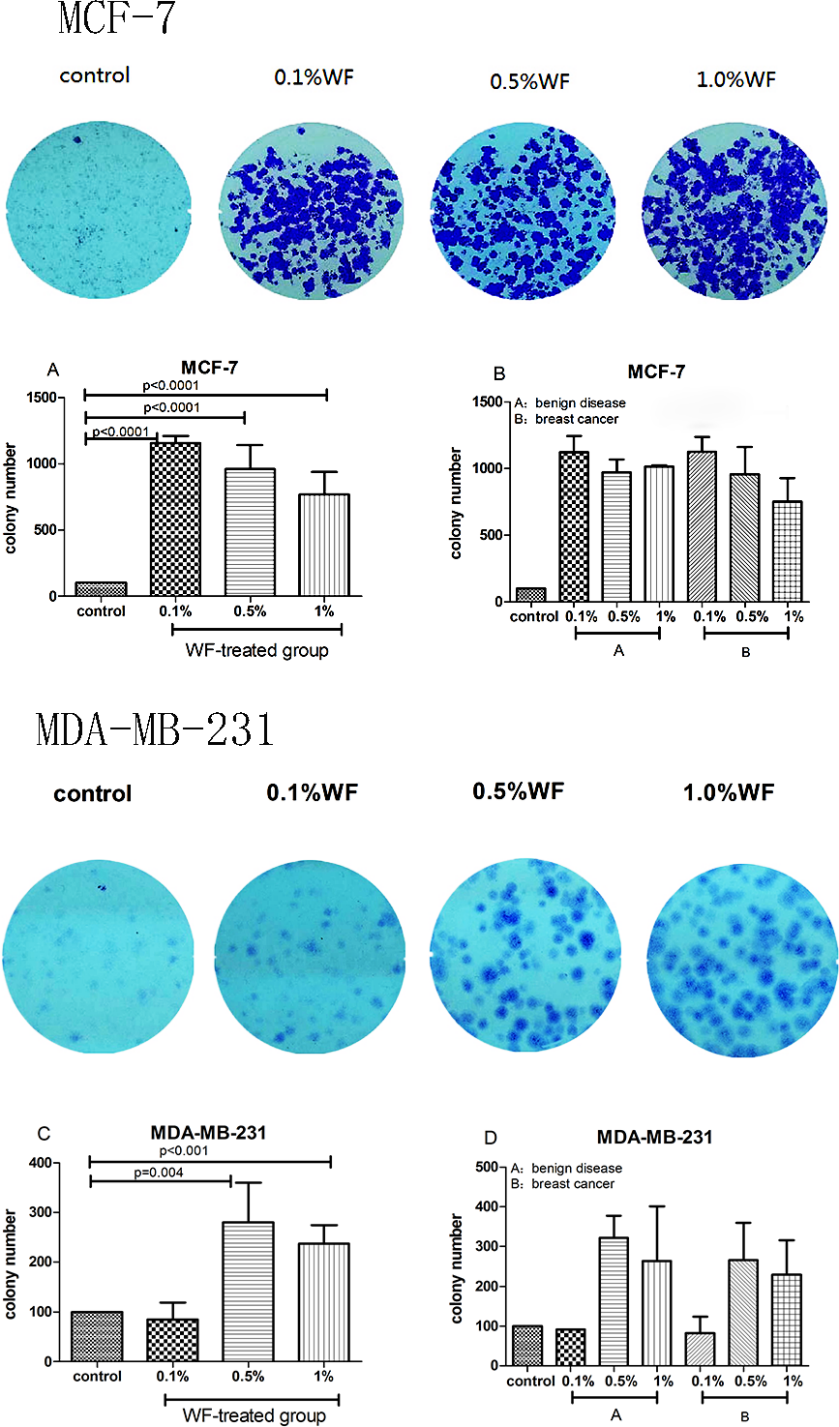
Colony formation assays: WF promotes MCF-7 and MDA-MB-231 cell growth Less colonies form (both MCF-7 and MDA-MB-231 cells) in the absence of WF compared to WF-treated groups. **A.** 0.1%, 0.5% and 1%WF promotes colony formation of MCF-7 cells. **C.** The numbers of colonies form by MDA-MB-231 cells in 0.5% and 1.0% WF-treated groups are significant higher than that in 0.1%WF-treated group and the control group. **B.** and **D.** No significant difference of colony numbers are observed between benign disease and breast cancer groups (both MCF-7 and MDA-MB-231 cells).

The results of colony formation assay are shown in Figure 1. The numbers of colonies formed by MCF-7 cells treated with WF (0.1%, 0.5%, and 1.0%) were significantly higher than that of the control group (Figure 1A, all *P* < 0.0001). Similarly, the numbers of colonies formed by MDA-MB-231 cells treated with WF (0.5%, and 1.0%) were significantly higher than that of the control group (Figure 1C, *P* = 0.004 and *P* < 0.001), while no difference was detected between cells cultured with 0.1% WF and solely medium. There were no significant differences found in the colony numbers between benign disease and breast cancer groups (both MCF-7 and MDA-MB-231, Figure 1B and Figure 1D).

**Figure 2:**
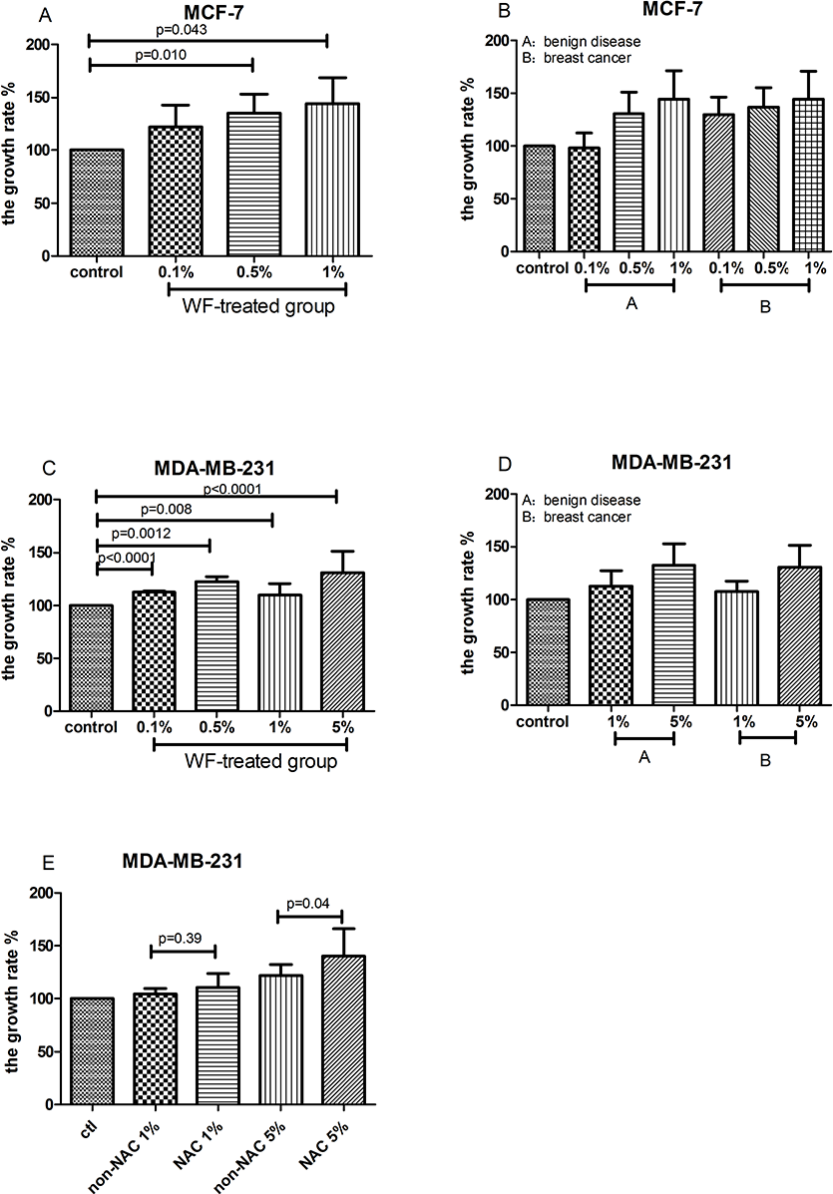
MTT proliferation assays: WF increases MCF-7 and MDA-MB-231 cell proliferation **A.** and **C.** The proliferation rates of MCF-7 and MDA-MB-231 cells are significantly increased after treated with WF for 48 hours when compared to the control group. **B.** and **D.** No significant difference of proliferation rate is observed between benign disease and breast cancer groups (both MCF-7 and MDA-MB-231 cells). **E.** MDA-MB-231 cells treated with WF from breast cancer patients underwent neoadjuvant chemotherapy (NAC) show higher rate of proliferation.

The MTT assay data was in accord with that obtained by performing in colony formation assays. The WF treated groups showed remarkable increase in proliferation when compared to the control cells (Figure 2). The proliferation rates of MCF-7 cells treated with 0.1%, 0.5% and 1.0% WF were 121.90% (±20.81%), 135.02% (±18.00%), and 138.58% (±27.66%), respectively, which were significantly higher than that of control group (in 0.5% and 1.0% WF, *P* = 0.010 and *P* = 0.043, respectively, Figure 2A). As of MDA-MB-231 cells, the proliferation rates in 0.1%, 0.5%, 1.0% and 5.0% WF were 112.6% (±1.35%), 122.60% (±4.78%), 110.04% (±10.52%) and 130.97% (±20.20%), respectively, which were also significantly higher when compared to that of untreated control cells (*P* < 0.0001, *P* = 0.0012, *P* = 0.008 and *P* < 0.0001, respectively, Figure 2C). No significant difference was detected in the proliferation rate between benign disease and breast cancer groups (both MCF-7 and MDA-MB-231, Figure 2B and 2D). Interestingly, MTT proliferation assay showed that MDA-MB-231 treated with WF from breast cancer patients underwent neoadjuvant chemotherapy (NAC) showed higher rate of proliferation compared to those treated with WF from patients without NAC (Figure 2E). Significant difference in proliferation rate was found between 5% WF and control groups (*P* = 0.04).

### Effects of WF on MDA-MB-231cell motility

**Figure 3:**
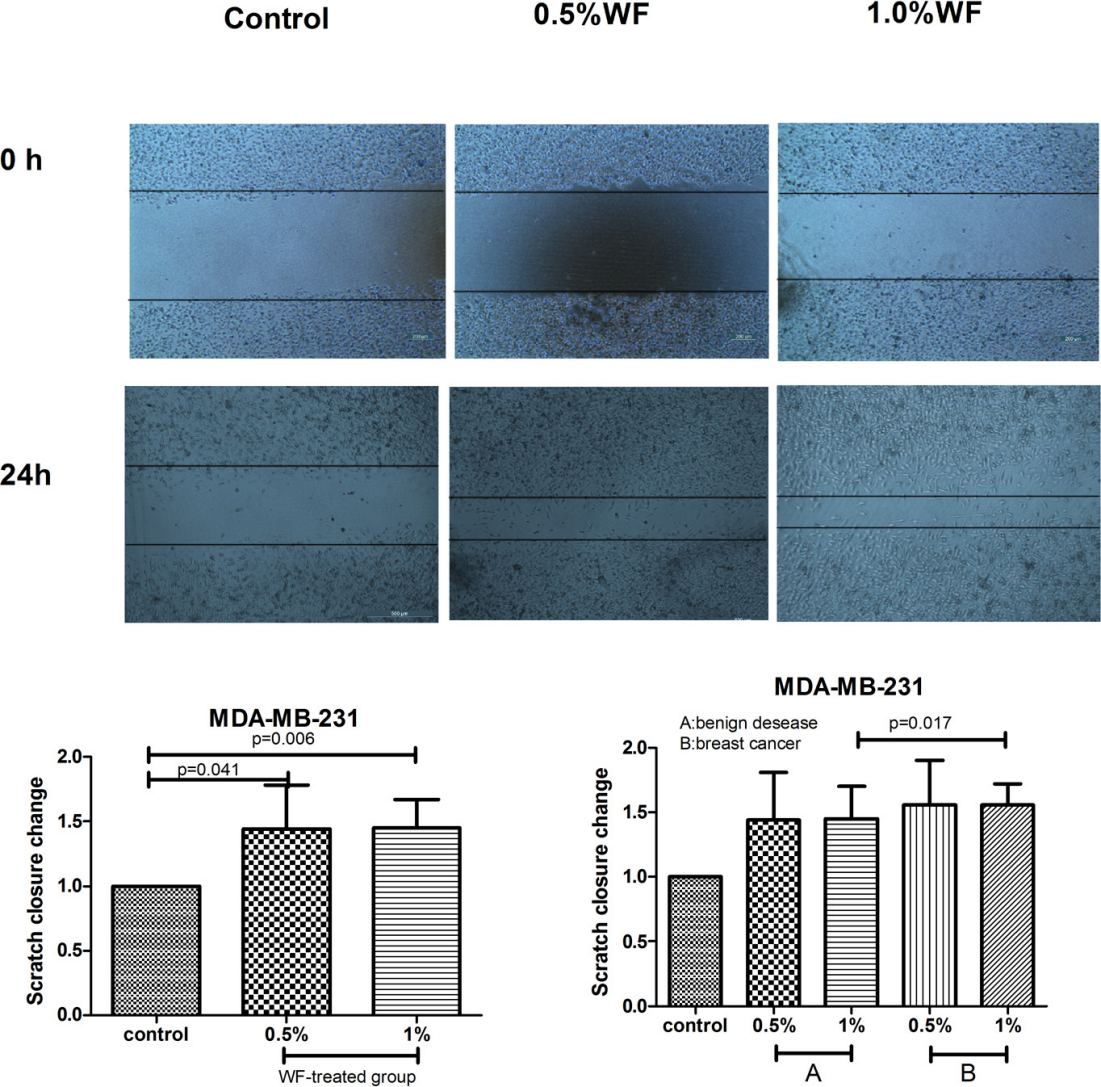
Scratch wounds assays: WF promotes MDA-MB-231cells migration Scratch wounds for MDA-MB-231 cells at 0 h and 24 h after introducing the wound and treated with WF are shown. Scratch closure change of control and WF groups for MDA-MB-231 cells was evaluated at 24 h. Scratch fold changes in 0.5% and 1% WF treated groups are significantly higher than that of the control group (*P* = 0.041 and *P* = 0.006). The scratch closure change of the cells treated with 0.5%WF was similar to that of 1% WF. Cells treated with 1% WF from breast cancer tend to migrate more rapidly than those treated with WF from benign disease (*P* = 0.017).

Scratch wound test was performed to assess the motility of MDA-MB-231cells. The migration of WF stimulated MDA-MB-231 cells was compared with the untreated control group (Figure 3). Cells in treated groups with 0.5% and 1% WF migrated more rapidly than that of the control groups and the differences were statistically significant (*P* = 0.041 and *P* = 0.006). Cells treated with 1% WF from breast cancer tended to recover more rapidly than that treated with WF from benign disease (*P* = 0.017).

### Quantitative and temporal profiles of biological factors in WF

Among the assessed 15 cytokines, 29 chemokines and 9 MMPs, the highest levels were found in IL-6, CCL21 and MMP-8, while the concentrations of IL-25, CCL11 and MMP-12 were the lowest. Furthermore, with the prolongation of postoperative period, the concentrations of IL-1β, IL-4, IL-6, IL-17F, IL-21, IL-23, IL-25, IL-31, IL-33, IFNγ and CD40L significantly decreased in the WF samples from breast cancer patients without NAC, as shown in Table 1.

**Table 1:** Significant differences in levels of cytokines in wound fluid in relation to post-surgery days

Cytokine	Group	*N*	*M* pg/ml	*X_25%_* pg/ml	*X_75%_* pg/ml	*P* value
IL-1β	Day 1	6	1541.37	370.31	2746.69	0.050
	Day 2	10	397.12	29.28	584.31	
	Day 3	12	149.44	31.03	939.91	
	Day 4	10	177.79	42.96	425.00	
IL-4	Day 1	6	27.09	20.24	38.16	0.006
	Day 2	10	16.55	12.83	19.71	
	Day 3	12	13.96	11.07	23.54	
	Day 4	10	11.38	8.12	17.52	
IL-6	Day 1	6	226176.00	170909.33	362337.25	0.003
	Day 2	10	118763.50	75960.74	216038.77	
	Day 3	12	78556.41	43517.65	160951.69	
	Day 4	10	46990.56	23458.15	96296.32	
IL-17F	Day 1	6	101.37	86.48	125.01	0.009
	Day 2	10	71.21	45.92	86.97	
	Day 3	12	50.28	33.91	75.20	
	Day 4	7	38.99	32.22	73.26	
IL-21	Day 1	6	1136.15	912.43	1521.65	0.003
	Day 2	10	604.90	349.05	855.75	
	Day 3	11	482.67	378.77	973.30	
	Day 4	9	340.79	204.17	576.16	
IL-23	Day 1	6	82.90	73.97	159.51	0.046
	Day 2	9	43.63	17.61	73.48	
	Day 3	7	40.00	10.77	70.48	
	Day 4	5	20.17	10.77	63.72	
IL-25	Day 1	6	16.15	13.17	26.48	0.004
	Day 2	10	11.59	5.06	14.00	
	Day 3	11	6.11	5.27	11.59	
	Day 4	9	4.41	1.62	7.11	
IL-31	Day 1	6	54.91	45.50	83.43	0.001
	Day 2	10	43.34	22.60	51.08	
	Day 3	12	27.54	22.60	38.99	
	Day 4	10	23.50	15.28	27.94	
IL-33	Day 1	6	213.63	44.66	296.57	0.003
	Day 2	10	55.65	33.48	113.35	
	Day 3	12	39.87	29.12	53.27	
	Day 4	10	23.80	17.77	30.94	
IFNγ	Day 1	6	210.72	184.53	288.92	0.011
	Day 2	8	145.83	85.98	197.05	
	Day 3	8	116.49	75.84	159.61	
	Day 4	7	98.64	44.67	128.45	
CD40L	Day 1	6	898.05	314.94	1322.21	<0.0001
	Day 2	10	301.67	141.66	339.24	
	Day 3	12	158.76	139.83	250.48	
	Day 4	10	130.35	94.26	174.03	

Levels are expressed as median and range in *X_25%_*, *X_75%_*.

Differences were considered to be significant at the *P* < 0.05 level. Some data was missing because the concentration was undetectable.

IL = Interleukin; IFNγ = Interferon γ; CD40L = CD40 ligand.

There was a significant variation in the concentrations of different cytokines among the samples with different biological features. Among the WF samples collected on the 2^nd^ day after surgery, those harvested from NAC patients showed significant increased levels of certain factors including IL-1β, IL-4, IL-6, IL-17F, IL-21, IL-23, IL-25, IL-31, INFγ, CD40L, TNFα, CXCL1, CXCL2, CXCL5, CCL3, CCL7 and CCL20 (Table 2). The concentrations of the following cytokines including IL-23, IL-25, IFNγ, CD40L and TNFα in WF collected from patients with T2 tumor tended to be higher than those with T1 tumor (Table 3). Similarly, CXCL5, CXCL13, CCL1, CCL7 and CCL26 in WF collected from patients with T1–2 tumor tend to be higher than those with Tis, and the profiles of CXCL13, CCL27, MMP-1 and MMP-7 in WF from N1–3 patients tend to be higher than those with N0 (Table 3). Meanwhile, only CCL2 showed a lower expression in WF from patients of N1–3 diseases. The levels of CX3CL1, CXCL1, CXCL5, CXCL6, CXCL11, CXCL12, CXCL13, CCL1, CCL3, CCL8, CCL11, CCL13, CCL20, CCL24, CCL25, CCL26, CCL27 and MMP-7 in WF harvested from patients with infiltrative mammary carcinoma tended to be higher than that from carcinoma *in situ* (Table 4). The concentration of MMP-12 in WF from breast cancer patients with luminal subtypes tended to be higher than that with HER2 overexpression and basal subtype (luminal A 703.41 pg/ml, luminal B 789.91 pg/ml, HER2 overexpression 436.47 pg/ml and basal 344.45 pg/ml, *P* = 0.013). There was no significant difference in the protein profiles between WF drained from chest wall and axillary wounds, neither was observed between benign and malignant diseases (Supplementary Table S4).

**Table 2:** Significant differences in levels of factors in wound fluid in relation to NAC

Factor	Group	*N*	*M* pg/ml	*X_25%_* pg/ml	*X_75%_* pg/ml	*P* value
IL-1β	Non-NAC	10	397.12	29.28	584.31	0.011
	NAC	7	1093.47	814.88	3555.27	
IL-4	Non-NAC	10	16.55	12.83	19.71	0.024
	NAC	7	21.15	18.46	28.27	
IL-6	Non-NAC	10	118763.50	75960.74	216038.77	0.015
	NAC	7	240568.27	213409.31	302916.58	
IL-17F	Non-NAC	10	71.21	45.92	86.97	0.002
	NAC	7	101.69	91.78	128.01	
IL-21	Non-NAC	10	604.90	349.05	855.75	0.008
	NAC	7	1113.52	875.29	1622.89	
IL-23	Non-NAC	9	43.63	17.61	73.48	0.003
	NAC	7	100.69	94.86	155.20	
IL-25	Non-NAC	10	11.59	5.06	14.00	0.010
	NAC	7	16.71	13.82	27.06	
IL-31	Non-NAC	10	43.34	22.60	51.08	0.036
	NAC	7	82.66	55.78	107.33	
IFNγ	Non-NAC	8	145.83	85.98	197.05	0.049
	NAC	7	212.37	158.29	335.37	
CD40L	Non-NAC	10	301.67	141.66	339.24	0.001
	NAC	7	602.84	486.55	808.91	
TNFα	Non-NAC	10	101.82	41.48	169.05	0.025
	NAC	7	223.80	149.13	232.31	
CXCL1	Non-NAC	23	834.00	394.76	2816.39	0.027
	NAC	8	2762.10	1920.99	5550.09	
CXCL2	Non-NAC	23	227.14	143.20	917.18	0.011
	NAC	8	1261.87	546.68	4006.32	
CXCL5	Non-NAC	23	3602.10	2132.47	17961.81	0.017
	NAC	8	32123.87	13323.82	52560.84	
CCL3	Non-NAC	23	173.66	53.85	515.78	0.047
	NAC	8	926.39	175.88	1051.18	
CCL7	Non-NAC	23	882.29	623.90	1111.82	0.030
	NAC	8	1574.15	923.87	2448.91	
CCL20	Non-NAC	23	3061.31	1526.12	5531.57	0.030
	NAC	8	7083.39	5023.03	9759.27	

Levels are expressed as median and range in *X_25%_*, *X_75%_*.

Differences were considered to be significant at the *P* < 0.05 level. Some data was missing because the concentration was undetectable.

IL = Interleukin; IFNγ = Interferon γ; CD40L = CD40 ligand; NAC = neoadjuvant chemotherapy; Non-NAC = never received neoadjuvant chemotherapy.

Cytokines and chemokines detection assays were two independent tests.

**Table 3:** Significant differences in levels of factors in wound fluid in relation to TNM stage

Factor	Group	*N*	*M* pg/ml	*X_25%_* pg/ml	*X_75%_* pg/ml	*P* value
IL-23	T1	14	36.08	10.77	78.30	0.015
	T2	22	82.90	45.39	100.69	
IL-25	T1	18	5.90	4.30	12.71	0.045
	T2	27	13.82	6.11	16.71	
IFNγ	T1	16	119.06	61.06	188.34	0.023
	T2	22	173.75	135.16	220.23	
CD40L	T1	19	161.67	129.61	294.33	0.028
	T2	28	320.08	145.66	633.10	
TNFα	T1	19	51.43	42.99	117.81	0.046
	T2	28	157.60	46.83	227.52	
CXCL5	Tis	3	1677.29	1280.93	-	0.034
	T1	15	7038.40	3517.02	34474.37	
	T2	13	10959.06	2560.28	37514.47	
CXCL13	Tis	3	13.48	7.48	-	0.035
	T1	15	20.64	16.75	28.76	
	T2	13	17.77	13.89	22.20	
CCL1	Tis	3	91.46	83.02	-	0.036
	T1	15	115.97	107.78	126.69	
	T2	13	112.20	107.78	120.43	
CCL7	Tis	3	621.35	425.28	-	0.032
	T1	15	836.33	649.01	946.68	
	T2	13	1398.15	925.17	1843.13	
CCL26	Tis	3	70.01	43.67	-	0.018
	T1	15	158.71	107.49	206.04	
	T2	13	121.24	98.04	147.95	
CXCL13	N0	19	16.40	12.45	20.64	0.047
	N1–3	12	21.80	17.90	28.61	
CCL2	N0	19	8387.95	7129.45	9713.62	0.023
	N1–3	12	6493.85	5841.05	7964.82	
CCL27	N0	19	868.50	416.13	1393.43	0.017
	N1–3	12	1727.04	1413.09	3228.77	
MMP-1	N0	19	19689.01	11580.62	49030.81	0.023
	N1–3	12	58166.49	31745.85	103371.65	
MMP-7	N0	19	22678.06	10194.38	35317.94	0.047
	N1–3	12	27746.61	23826.58	57473.62	

Levels are expressed as median and range in *X_25%_*, *X_75%_*. Differences were considered to be significant at the *P* < 0.05 level. Some data was missing because the concentration was undetectable.

IL=Interleukin; IFNγ = Interferon γ; CD40L = CD40 ligand; TNFα = tumor necrosis factor α MMPs = matrix metalloproteinase.

Cytokines, chemokines and MMPs detection assays were independent tests.

**Table 4:** Significant differences in levels of factors in wound fluid in relation to pathological type

Factor	Group	*N*	*M* pg/ml	*X_25%_* pg/ml	*X_75%_* pg/ml	*P* value
CX3CL1	CIS	7	1250.66	803.87	1305.94	0.021
	IBC	24	2003.09	1448.02	2304.44	
CXCL1	CIS	7	377.41	282.22	1155.54	0.004
	IBC	24	2202.03	805.26	4051.58	
CXCL5	CIS	7	1801.58	1280.93	3517.02	0.005
	IBC	24	11529.00	3198.47	41240.20	
CXCL6	CIS	7	69.23	56.35	78.94	0.004
	IBC	24	100.04	81.32	159.62	
CXCL11	CIS	7	16.76	9.67	25.72	0.047
	IBC	24	34.30	16.14	48.73	
CXCL12	CIS	7	1645.80	1353.16	1695.97	0.021
	IBC	24	2161.32	1720.48	2604.57	
CXCL13	CIS	7	13.48	8.89	20.64	0.032
	IBC	24	18.97	16.27	28.08	
CCL1	CIS	7	91.46	83.02	107.78	0.004
	IBC	24	114.10	109.46	125.59	
CCL3	CIS	7	53.60	18.67	240.66	0.030
	IBC	24	222.52	141.28	1026.69	
CCL8	CIS	7	69.63	46.99	105.36	0.033
	IBC	24	103.27	86.48	396.57	
CCL11	CIS	7	26.61	22.24	27.92	0.001
	IBC	24	31.74	28.99	34.79	
CCL13	CIS	7	37.03	27.61	44.50	0.003
	IBC	24	76.04	63.15	116.34	
CCL20	CIS	7	1792.22	271.21	2569.68	0.018
	IBC	24	4969.35	2221.55	8682.70	
CCL24	CIS	7	123.99	83.27	283.46	0.023
	IBC	24	574.51	193.14	877.40	
CCL25	CIS	7	2006.27	1922.35	2391.63	0.038
	IBC	24	2514.82	2075.52	3030.91	
CCL26	CIS	7	70.01	67.96	84.47	0.003
	IBC	24	140.44	108.67	195.93	
CCL27	CIS	7	619.63	267.41	1178.84	0.018
	IBC	24	1507.12	852.57	3076.87	
MMP-7	CIS	7	15855.12	9792.97	22678.06	0.023
	IBC	24	26771.56	21489.70	44569.89	

Levels are expressed as median and range in *X_25%_*, *X_75%_*. Differences were considered to be significant at the *P* < 0.05 level.

MMPs = matrix metalloproteinase; IBC = Invasion breast cancer; CIS = Carcinoma *in situ*. Chemokines and MMPs detection assays were different tests.

## DISCUSSION

In this work, proliferation data obtained in colony formation assay and MTT assay revealed that WF treatment significantly induced breast cancer cells proliferation, which agrees with previous studies [19, 20]. Furthermore, in cells treated with WF from patients underwent NAC, the stimulative proliferation was more remarkable. Migration data obtained from scratch wound assay confirmed the stimulatory effect of WF on breast cancer cell motility. These findings support the hypothesis that surgical wounds can modify the tumor microenvironment, induce tumor growth and promote local recurrence by stimulating the proliferation and motility of residual cancer cells [21–23]. To our best knowledge, this is the first study describing the increased levels of cytokines related to inflammatory and chemokines in WF due to NAC. Also, the WF composition test revealed that the levels of cytokines, chemokines, and MMPs varied within different tumor staging and pathological type groups.

Surgery is one of the worldwide accepted standard procedures for breast cancer treatment; however, surgical wound may induce a multifactorial wound healing process including inflammation, neovascularization, and matrix deposition and reorganization. Previous research indicated that the surgical wound-induced factors might be associated with the activation of tumor-initiating cells, which share properties of self-renewal and differentiation with normal stem cells [11]. These evidences on surgical wounds provide an explanation that “residual tumor cell” may persist within negative excision margins and mediate local recurrence in the bed of primary tumors several years post-surgery.

Mastectomy leaves an acute wound that the residual tumor cells expose to the WF automatically, which increases the risk of recurrence. Therefore, the composition of WF and whether the components will contribute to cancer progression are of clinical interests. Our study investigated the temporal and quantitative profiles of T-helper cell 17-type response pathway related 15 cytokines, 29 chemokines and 9 MMPs in WF after mastectomy. The concentrations of IL-1β, IL-4, IL-6, IL-17F, IL-21, IL-23, IL-25, IL-31, IL-33, INFr and CD40L reversely correlated with the prolongation of postoperative period in samples from breast cancer patients without NAC. The process of wound healing induces by acute tissue injury is commonly divided into three overlapping stages [24]. The first inflammatory stage is coagulation by activation of platelets, which release growth factors and chemokines. In response to these chemokines, lymphocytes and leukocytes enter into the wound within hours during the second stage, followed by the secretion of interleukins which mediate the biological function including fibroblast proliferation, ECM remodeling and angiogenesis at day 3–4 during the third stage. These remarks completely confirm our observations.

There was a significant variation in the concentrations of different inflammatory related cytokines and chemokines among the samples with different biological features. Interestingly, the WF harvested from NAC patients showed significant increased expression of IL-1β, IL-4, IL-6, IL-17F, IL-21, IL-23, IL-25, IL-31, IFNγ, CD40L, TNFα, CXCL1, CXCL2, CXCL5, CCL3, CCL7 and CCL20. To our best knowledge, this is the first study describing the increased levels of cytokines related to inflammatory and chemokines in WF due to NAC. Chemotherapy may induce upgraded local inflammation after surgery. We suggest that chemotherapy itself causes tumor cell deaths and leads to cell necrosis that introducing an environment rich of reactive oxygen species (ROS). Furthermore, the surgical trauma may aggravate the phenomenon by activating Nox enzymes and pro-inflammatory mediators. The resulting redox signaling may promote cancer cell invasion, adhesion, and metastasis [25]. Nonetheless, the roles of the cytokines and chemokines in tumor progression during wound healing are barely known, regardless of in response to NAC.

Biological factors produced by the primary tumor are implicated in the formation of the pre-recurrence niche by homing of disseminated tumor cells and pre-metastatic niche by the recruitment of bone marrow derived cells (BMDC) and by remodelling of the extracellular matrix [26, 27]. Several chemokines, cytokines and MMPs have been proved to be closely related to the degree of malignancy. In our study, the concentrations of IL-23, IL-25, IFNγ, CD40L, TNFα, CXCL5, CXCL13, CCL1, CCL7, CCL26, CCL27, MMP-1 and MMP-7 in WF were in parallel with the TNM stage of tumor. In addition, the levels of CX3CL1, CXCL1, CXCL5, CXCL6, CXCL11, CXCL12, CXCL13, CCL1, CCL3, CCL8, CCL11, CCL13, CCL20, CCL24, CCL25, CCL26, CCL27 and MMP-7 in WF harvested from patients with infiltrative mammary carcinoma tended to be higher than that from carcinoma *in situ*. The above evidences indicate that these factors may contribute to the degree of malignancy and the crosstalk between cancer and host cells could be mediated by them. Our study presented that CCL2 had a negative correlation with lymph node staging; however, its role in tumor prognosis has not yet been determined. Likewise, MMP-12 could be a benign prognosis index, because the concentrations of MMP-12 in WF from the breast cancer patients with luminal subtypes tended to be higher than that with HER2 overexpression and basal subtypes.

It is well known that IL-6 family belong to proinflammatory cytokines secreted by fibroblast cells, macrophages and neutrophils. Members in this family help to promote cancer progression via the following three pathways: JAK-STAT3, SHP-2-Ras-EPK cascade and PI3K-Akt pathways [28, 29]. Previous research also indicated that increased resistance to apoptosis in human breast cancer was induced by high expression of IL-6 and IL-6R [30]. IL-4, a Th2 cytokine, has been showed increased in the microenvironment of breast carcinomas [31], and IL-4R was overexpressed by breast cancer cells themselves [32]. The IL-4/IL-4R interaction enhanced the proliferation and survival of breast cancer cells *in vitro* [33, 34]. A recent report showed that the type II IL4R was expressed and activated in human breast cancer and the metastatic capacity was decreased by knocking down IL4Rα, therefore inactivating Erk1/2, Akt and mTor induced reduction in breast cancer proliferation and survival [35]. IL-17 is a proinflammatory cytokine most prominently produced by T-helper type 17 (Th17) cells and frequently expresses in multiple cancers, including breast cancer [36]. IL-17 promotes tumor proliferation, survive and metastasis by up regulating angiogenesis factors VEGF, CXCL8, MMP2 and MMP9 [37], while at the same time, it significantly suppresses apoptosis in several human breast carcinoma cell lines, such as 4T1, MDA-MB-231 cells. The knockdown of IL-17R in 4T1 mouse mammary cancer cells enhanced apoptosis and decreased tumor growth [38]. IL-25, a proinflammatory cytokine, secreted by normal mammary epithelial cells and induces apoptosis of tumor cells via IL-25R (IL-17RB) highly expressed in tumor cells and barely expressed in normal epithelial cells [39]. Overexpression of IL-25R on the surface of malignant, mammary cells correlated with tumorigenic potential of breast cancer cells and poor prognosis [39, 40], while IL-25/IL-25R pathway resulted in specific apoptosis in breast cancer cells [40]. IL-1β and TNFα are produced by macrophages, monocytes and other cells of innate immune system in response to harmful stimuli such as cytotoxic agents [41], which confirms our observation of increased IL-1β and TNFα in NAC patients. Furthermore, TNFα increased proliferation of human breast cancer cell line T47D through the intracellular signaling p42/p44 MAPK, JNK, PI3-K/Akt pathways and NF-kappa B transcriptional activation trigged by TNFα/TNFR1, TNFR 2 pathways [42].

The expression of interferon γ (IFNγ) in tumors improves tumor specific T cell recruitment and mediates the apoptosis of breast cancer cells via down-regulation of anti-apoptosis Bcl-2 family members [43] and inducing growth arrest at mid-G1. Previous study indicated that IFNγ and IFNγ-Rα immunoreactions presented in the cytoplasm, while IFNγ-Rβ was also found in the nucleus [44]. CD40, a TNF receptor family member, is expressed in B-lymphocytes, macrophages, fibroblasts, endothelial and epithelial cells and its ligand CD40L was proved to express in breast tumor. Co-expression of CD40 and CD40L contributes to oncogenic process of malignancy *in vitro*, increasing tumor proliferation, motility and invasion by activation CD40L/CD40/NF-jB pathway [45].

Chemokines CXCL1, CXCL2 and CXCL12 have been proved to play important roles in the growth of various cancers by activating MAPK/ERK signaling pathway and thus promote tumor cell proliferation [46, 47]. The CXCR4/CXCL12 axis has been thoroughly investigated and these two factors are upregulated in breast cancer cell lines as well as the most common sites of breast cancer metastasis [48]. Moreover, the metastasis of breast tumor cells to the lymph nodes and lungs was significantly decreased by inhibiting CXCR4-CXCL12 interactions *in vivo* [48]. CCR7 mediated leukocyte migration in normal immune responses by two chemokine ligands: CCL19 and CCL21. Prior publications reported that a majority of primary breast cancer tissues and metastatic cancer cells in the lymph nodes overexpress CCR7. Furthermore, higher CCR7 expression is correlated with compromised survival in breast cancer patients [49]. CXCR2 and its ligand CXCL1 contribute to the resistance of chemotherapy in mammary tumor cells and the knockdown of CXCR2 enhances sensitivity to chemotherapy and inhibits tumor cell metastasis [50, 51]. The CCR5 axis participates in breast cancer cells invasion, serving as a driver for metastasis and recruiting specific immune cells into tumors, inducing local immunosuppression and contributing to tumor progression [52]. CXCL13–CXCR5 co-expression regulates epithelial to mesenchymal transition of breast cancer cells during lymph node metastasis in infiltrating duct carcinoma [53].

It has been reported that MMP-1 increased migration and invasion of breast cancer cell due to slaving and activating the protease activated receptor-1 (PAR-1) [54]. MMP-3 or MMP-7 generates a bioactive fragment that promotes invasion and Epithelial-Mesenchymal Transition (EMT) in mammary epithelial cells by targeting of E-cadherin [55].

In conclusion, our findings demonstrated that post-surgery WF promotes the proliferation and migration of breast cancer cells and that the proliferative effect is concentration-dependent to a certain extent, which supports the previous findings that surgery may have adverse effects on breast cancer patients. Characterizing potential therapeutic targets in WF to inhibit further breast cancer proliferation is of clinical interest in future researches. However, this work still has some limitations. For instance, *in vivo* study has not yet been carried out to verify current results. Moreover, endeavor is needed to pay in clearly illustrating the crosstalk among the components in WF as well as their roles in wound healing process and tumor progression. Elucidating the molecular pathways through which WF mediates breast cancer development can lead to the discovery of novel methods to control local recurrence and metastasis by introducing management strategies.

## MATERIALS AND METHODS

### Patients and wound fluid collection

A total of 72 wound fluid (WF) samples were collected from a cohort of 42 patients underwent mastectomy with axillary dissection for breast cancer and 3 patients with mammary benign disease surgeries between September and December, 2014 at The Second Hospital of Dalian Medical University, China. All patients had no underlying diseases except breast neoplasm. A flow chart with the information of all WF samples (including the number of patients) for all tests is listed in Supplementary Figure S1. Twenty-four WF samples from 24 patients were enrolled for the cell co-culture study (Supplementary Table S1), 49 WF samples from 25 patients were for cytokines test (Supplementary Table S2), and 34 samples from 34 patients were for chemokines and MMPs test (Supplementary Table S3). The WF samples in each independent study were partial repeated. Drainage WF was collected from patients at post-surgery day 1 to 4. The perforated end of the surgical drain was placed in the chest wall and/or axilla wounds. Each 50 mL WF sample was collected in a sterile container without additives, centrifuged at 1, 600 RCF for 10 minutes, and then the supernatant was separated into 25 shares (2 mL per share) and stored frozen in sterile freezing tube at −80°C. Written informed consent was obtained from individual patients, and the experimental protocol was approved by the Ethics Committee of Dalian Medical University.

### Cell culture

WF samples were thawed on the ice and centrifuged at 14, 000 RCF for 10 minutes to remove cell debris before each independent study. The samples were then passed through a 0.22-μm filter to remove bacterial and used for subsequent cell culture studies *in vitro*. Nine WF samples were prepared for assessing the effect of WF on MCF-7 (luminal, ER+, PR+, HER2^_^) and 21 samples were co-cultured with MDA-MB-231 (basal-like, ER^_^, PR^_^, HER2^_^) for the same purpose. Both breast cancer cell lines were obtained from American Type Culture Collection (ATCC) and cultured in Dulbecco's modified Eagle's medium (DMEM, SIGMA, USA) supplemented with 10% fetal bovine serum (FBS, SIGMA, USA) and 1% penicillin/streptomycin (P/S, Gibco, USA). The characteristics of WF samples were listed in Supplementary Table S1.

### Colony formation test

The MCF-7 and MDA-MB-231 cells were plated in 12-well plates (5 × 10^3^/ml) with 1ml DMEM containing 10% FBS and then incubated at 37°C for 24 hours. The culture medium was replaced with DMEM containing 2% FBS and supplemented with different levels of WF (0.1%, 0.5% or 1%) for the following 2 weeks. For the negative control, medium was replaced with DMEM only containing 2% FBS. The culture plates were washed twice with phosphate-buffered saline (PBS), fixed with 1% paraformaldehyde for 10 minutes and stained with crystal violet right before clony formation test. The stained cells were dissolved with 10% glacial acetic acid and the colony numbers were assessed using a microplate reader (Titertek Multiskan PLUS, MK II, Labsystems, USA) set at 595 nm.

### MTT cell proliferation test

The proliferation potential of MCF-7 and MDA-MB-231 cells was tested by MTT assay. MCF-7 and MDA-MB-231cells (5 × 10^4^/ml) were seeded onto 96-well plates in 100 μL DMEM containing 10% FBS. After incubation for 24 hours, the culture medium was replaced with DMEM containing 2% FBS supplemented for both WF-treated (0.1%, 0.5% and 1% WF) and control groups at 37°C. 10 μL sterile MTT dye/well was added to each well after 48 hours of co-culture and incubated at 37°C for 4 hours. The MTT solution was removed and 200 μL of dimethyl sulfoxide (DMSO) was added to dissolve the formazan crystals. The absorbance was measured at 570 nm using a microplate reader (Titertek Multiskan PLUS, MK II, Labsystems, USA).

### Scratch wound assay test

Vertical lines were drawn across the wells at the back of the 24-well plates using a marker pen. MDA-MB- 231 cells with 90%-100% confluence were plated uniformly in 18 wells of the 24-well plates in 500 μl DMEM containing 10% FBS for the consistent cellular density upon the previous experiments. The scratch was placed on the next day. Lines perpendicular and parallel to the vertical lines (equal to a“+”) were employed with the tip of a 200 μl pipette in the presence of the original medium at the bottom. The cells were washed with PBS (1 ml/well) and the sloughing cells were removed. Initial cell status was visualized immediately using a microscope (Leica DM4000B, Germany). Then the medium was replaced with 500 μl DMEM containing 2% FBS for both WF-treated (0.5% and 1% WF) and control groups. Images of scratch wounds were acquired after 24 hours co-incubated with WF. Scratch closure rate was measured using an image processing software (Image J, NIH, USA). The area between cells was measured from 2 different regions on a single scratch.

### Wound fluid composition analysis

Forty-nine and 34 samples were collected to investigate the quantitative and temporal profiles of cytokines and chemokines/MMPs respectively, which were expected to be present in WF. The former samples were assayed by Bio-Plex Pro TM human Th17 cytokine kit (Bio-Rad Laboratories, USA) based on magnetic bead that detected 15 cytokines (IL-1β, IL-4, IL-6, IL-10, IL-17A, IL-17F, IL-21, IL-22, IL-23, IL-25, IL-31, IL-33, IFNγ, CD40L, TNFα). Similarly, the latter samples were assayed by Bio-Plex Pro human 29 chemokines (Bio-Rad Laboratories, USA, CCL1, CCL2, CCL3, CCL7, CCL8, CCL11, CCL13, CCL15, CCL17, CCL19, CCL20, CCL21, CCL22, CCL23, CCL24, CCL25, CCL26, CCL27, CXCL1, CXCL2, CXCL5, CXCL6, CXCL9, CXCL10, CXCL11, CXCL12, CXCL13, CXCL16 and CX3CL1) and Bio-Plex ProTM human MMPs (Bio-Rad Laboratories, USA, MMP-1, MMP-2, MMP-3, MMP-7, MMP-8, MMP-9, MMP-10, MMP-12, MMP-13). The assays are immunoassays similar to sandwich ELISA that the capture antibodies couple with magnetic bead and biotinylated detection antibodies couple with streptavidin-phycoerythrin conjugate fluorescent reporter which reacts with the sample containing biomarker of interest. The expression of each cytokine was calculated by densitometric analysis of fluorescent indicator using Bio-Plex 200 reader (Bio-Rad Laboratories, USA) and the data were presented by Bio-Plex Manager TM software as median fluorescence intensity and concentration (pg/ml). Biological factor expression was normalized using the positive controls presented in the array.

### Statistical analysis

All statistical analyses were performed using the SPSS statistics 16.0 software package (SPSS Inc., Chicago, USA). A *p*-value of < 0.05 was considered statistically significant. The computer program PRISM (version 5; GraphPad Inc., USA) and Image J (NIH, USA) were used to create graphs, process images and perform statistical analysis. The independent *T*-test (cell co-culture tests) and Mann-Whitney test (WF composition analysis) were applied to evaluate the differences between unrelated groups. Paired *T*-test and Wilcoxon test were used to assess differences between WF samples drained from chest wall and axillary wounds. The Kruskal-Wallis H test was employed to evaluate discordances in the concentrations of various cytokines during the prolongation of postoperative period.

## SUPPLEMENTARY FIGURE AND TABLES


