# Supplementary material for: High throughput screening of cytokines, chemokines and matrix metalloproteinases in wound fluid induced by mammary surgery
Source: Oncotarget. 2015 Aug 10;6(30):29296–310. doi: 10.18632/oncotarget.4828 (PMC4745727; doi:10.18632/oncotarget.4828)
Supplement: Supplementary file 1 [file oncotarget-06-29296-s001.pdf]

SUPPLEMENTARY FIGURE AND TABLES

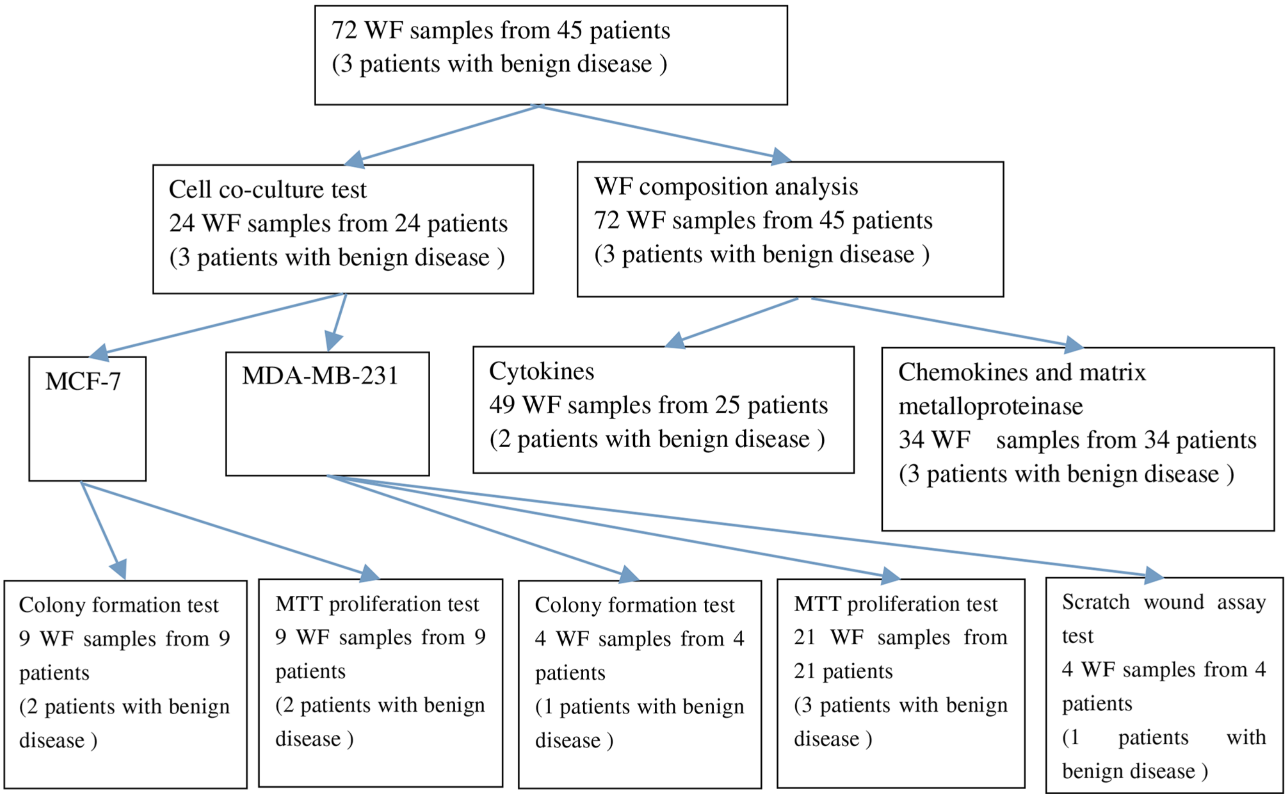

Supplementary Figure S1: The total WF samples and their source for all tests.

Supplementary Table S1: Main characteristics of WF samples for cell co-culture test

Characteristics of samples		No.			
Co-culture with		MCF-7		MDA-MB-231	
WF from-					
Benign disease		2		3	
Breast cancer		7		18	
neoadjuvant chemotherapy					
+		3		8	
Therapy regimen	CEF		0		2
	TE		3		4
	TC		0		2
−		4		10	
T stage					
T1		1		7	
T2		6		11	
N stage					
N0		2		9	
N1–3		5		9	
Pathological type					
Invasion breast cancer		7		17	
Carcinoma in situ		0		1	
Pathological stage					
II		7		15	
III				2	
Molecular subtype					
Luminal A		2		2	
Luminal B		2		8	
HER2 overexpression		1		4	
Basal-like		2		2	

(1) 24 WF samples from 24 patients were enrolled for the cell co-culture study. Among three patients with benign disease, two patients were diagnosed as mammary fibroadenosis and one as intraductal papilloma.
(2) There were two patients in MDA-MB-231 co-culture test not accepted immunohistochemistry test for tumor subtype assay.
(3) CEF = Cyclophosphamide/Epirubicin/5-Fluorouracil
TE = Docetaxel/Epirubicin
TC = Docetaxel/Cyclophosphamide

Supplementary Table S2: Main characteristics of 49 samples from 25 patients for WF composition of cytokines test

Characteristics of patients		No.	%	Characteristics of WF samples		No.	%
WF from-				WF from-			
Benign disease		2	8.00	Benign disease		2	4.08
Breast cancer		23	92.00	Breast cancer		47	95.92
Breast cancer patients				Breast cancerWF samples			
Age at surgery, years				The collection time, days after surgery(without NAC)			
≤ 45		8	34.78	1		6	15.78
> 45		15	65.22	2		10	26.32
NAC				3		12	31.58
+		4	17.39	4		10	26.32
Therapy regimen	CEF	2		Age at surgery, years			
	TE	2		≤ 45		18	38.30
–		19	82.61	> 45		29	61.70
T stage				NAC(postoperative day 2)			
T1		9	39.13	+		7	41.18
T2		14	60.87	Therapy regimen	CEF	3	
N stage					TE	4	
N0		15	65.22	–		10	58.82
N1–3		8	34.78				
Pathological type				T stage			
Invasion breast cancer		19	82.61	T1		19	40.43
Carcinoma in situ		4	17.39	T2		28	59.57
Pathological stage				N stage			
II		16	84.21	N0		33	70.21
III		3	15.79	N1–3		14	29.79
Molecular subtype				Drainage tube location			
Luminal A		4	18.18	Chest well		20	42.55
Luminal B		11	50.00	axilla		22	46.81
HER2 overexpression		3	13.64	Mix		5	10.64
Basal-like		4	18.18				

(1) The two lenign diseaase patients were diagnosed as mammary fibroadenosis.
(2) There was one patient not accepted immunohistochemistry test for tumor subtype assay.
(3) NAC = Neoadjuvant chemotherapy
CEF = Cyclophosphamide/Epirubicin/5-Fluorouracil
TE = Docetaxel/Epirubicin

Supplementary Table S3: Main characteristics of 34 samples from 34 patiens for WF composition of chemokines and matrix metalloproteinases tests

Characteristics		No.		%
WF from-				
Benign disease		3		8.82
Breast cancer		31		91.18
WF samples from breast cancer patients				
Age at surgery, years				
≤ 45		8		25.81
> 45		23		74.19
Neoadjuvant chemotherapy				
+		8		25.81
Therapy regimen	CEF		2	
	TE		4	
	TC		2	
–		23		74.19
T stage				
Tis		3		9.68
T1		15		48.39
T2		13		41.93
N stage				
N0		19		61.29
N1–3		12		38.71
Pathological type				
Invasion breast cancer		24		77.42
Carcinoma in situ		7		22.58
Pathological stage				
I		1		4.17
II		22		91.66
III		1		4.17
Molecular subtype				
Luminal A		6		22.22
Luminal B		15		55.56
HER2 overexpression		4		14.81
Basal-like		2		7.41

(1) Among the three lenign diseaase patients, two patients were diagnosed as mammary fibroadenosis and one as intraductal papilloma.
(2) There were four patients not accepted immunohistochemistry test for tumor subtype assay.
(3) CEF = Cyclophosphamide/Epirubicin/5-Fluorouracil
TE = Docetaxel/Epirubicin
TC = Docetaxel/Cyclophosphamide

Supplementary Table S4: The differences of various factors' concentration in WF between benign disease and breast cancer groups

Biological factor	group	*N*	Median (pg/ml)
IL-1β	Benign disease	2	336.17
	Breast cancer	47	314.00
IL-4	Benign disease	2	24.39
	Breast cancer	47	17.52
IL-6	Benign disease	2	128039.81
	Breast cancer	47	127931.81
IL-10	Benign disease	2	138.83
	Breast cancer	47	251.33
IL-17A	Benign disease	2	41.92
	Breast cancer	47	37.84
IL-17F	Benign disease	2	84.90
	Breast cancer	44	74.26
IL-21	Benign disease	2	818.50
	Breast cancer	45	663.69
IL-22	Benign disease	2	8.51
	Breast cancer	35	12.39
IL-23	Benign disease	2	37.42
	Breast cancer	36	57.37
IL-25	Benign disease	2	12.97
	Breast cancer	45	11.22
IL-31	Benign disease	2	55.49
	Breast cancer	47	42.04
IL-33	Benign disease	2	270.56
	Breast cancer	47	42.75
IFNɤ	Benign disease	2	184.43
	Breast cancer	38	156.29
CD40L	Benign disease	2	387.73
	Breast cancer	47	234.39
TNFα	Benign disease	2	30.31
	Breast cancer	47	109.24
CCL1	Benign disease	3	103.13
	Breast cancer	31	112.2
CCL2	Benign disease	3	8626.73
	Breast cancer	31	7490.56
CCL3	Benign disease	3	239.52

(*Continued*)

Biological factor	group	*N*	Median (pg/ml)
	Breast cancer	31	216.17
CCL7	Benign disease	3	1377.59
	Breast cancer	31	890.49
CCL8	Benign disease	3	71.39
	Breast cancer	31	101.18
CCL11	Benign disease	3	27.56
	Breast cancer	31	28.89
CCL13	Benign disease	3	50.29
	Breast cancer	31	65.42
CCL15	Benign disease	3	1691.70
	Breast cancer	30	929.25
CCL17	Benign disease	3	182.09
	Breast cancer	31	184.79
CCL19	Benign disease	3	499.75
	Breast cancer	31	386.17
CCL20	Benign disease	3	1867.66
	Breast cancer	31	3611.83
CCL21	Benign disease	2	436908.53
	Breast cancer	29	96973.60
CCL22	Benign disease	3	3005.85
	Breast cancer	31	1238.80
CCL23	Benign disease	3	289.94
	Breast cancer	31	265.41
CCL24	Benign disease	3	315.07
	Breast cancer	31	378.55
CCL25	Benign disease	3	2025.61
	Breast cancer	31	2391.63
CCL26	Benign disease	3	102.23
	Breast cancer	31	124.43
CCL27	Benign disease	3	581.84
	Breast cancer	31	1354.17
CXCL1	Benign disease	3	1342.78
	Breast cancer	31	1330.81
CXCL2	Benign disease	3	395.16
	Breast cancer	31	478.36
CXCL5	Benign disease	3	5360.75
	Breast cancer	31	7038.40

(*Continued*)

Biological factor	group	*N*	Median (pg/ml)
CXCL6	Benign disease	3	83.89
	Breast cancer	31	96.24
CXCL9	Benign disease	3	585.15
	Breast cancer	29	901.81
CXCL10	Benign disease	3	417.04
	Breast cancer	31	472.05
CXCL11	Benign disease	3	11.66
	Breast cancer	31	25.68
CXCL12	Benign disease	3	2161.32
	Breast cancer	31	2004.96
CXCL13	Benign disease	3	19.75
	Breast cancer	31	18.27
CXCL16	Benign disease	3	1066.81
	Breast cancer	31	994.43
CX3CL1	Benign disease	3	1431.39
	Breast cancer	31	1862.78
MMP-1	Benign disease	3	52166.35
	Breast cancer	31	34161.72
MMP-2	Benign disease	3	474000.92
	Breast cancer	31	221828.01
MMP-3	Benign disease	3	150125.86
	Breast cancer	31	190171.94
MMP-7	Benign disease	3	71996.59
	Breast cancer	31	24602.15
MMP-8	Benign disease	3	399455.46
	Breast cancer	31	446213.40
MMP-9	Benign disease	3	275231.39
	Breast cancer	31	305829.68
MMP-10	Benign disease	3	15114.81
	Breast cancer	31	16106.90
MMP-12	Benign disease	3	900.67
	Breast cancer	31	690.04
MMP-13	Benign disease	3	1104.80
	Breast cancer	31	947.03
